# Photoacclimation and induction of light-enhanced calcification in the mesophotic coral *Euphyllia paradivisa*

**DOI:** 10.1098/rsos.180527

**Published:** 2019-02-06

**Authors:** Gal Eyal, Itay Cohen, Lee Eyal-Shaham, Or Ben-Zvi, Yaron Tikochinski, Yossi Loya

**Affiliations:** 1 School of Zoology, Tel-Aviv University, Tel Aviv 69978, Israel; 2 The Interuniversity Institute for Marine Sciences of Eilat, Eilat 88103, Israel; 3 The Steinhardt Museum of Natural History, Israel National Center for Biodiversity Studies, Tel Aviv 69978, Israel; 4 The Institute of Earth Sciences, The Hebrew University of Jerusalem, Jerusalem 9190401, Israel; 5 School of Marine Sciences, Ruppin Academic Center, Michmoret 40297, Israel

**Keywords:** coral reefs, mesophotic coral ecosystems, Red Sea, photosynthesis, light-enhanced calcification, twilight zone

## Abstract

Corals and their photosymbionts experience inherent changes in light along depth gradients, leading them to have evolved several well-investigated photoacclimation strategies. As coral calcification is influenced by light (a process described as LEC—‘light-enhanced calcification’), studies have sought to determine the link between photosynthesis and calcification, but many puzzling aspects still persist. Here, we examine the physiology of *Euphyllia paradivisa*, a coral species found at a wide range of depths but that is strictly mesophotic in the Red Sea; and also examines the coupling between photosynthesis and LEC by investigating the response of the coral under several controlled light regimes during a long-term experiment. *E. paradivisa* specimens were collected from 40 to 50 m depth and incubated under three light conditions for a period of 1 year: full-spectrum shallow-water light (approx. 3 m, e.g. shallow-light treatment); blue deep-water light (approx. 40 m, e.g. mesophotic-light treatment) or total darkness (e.g. dark treatment). Net photosynthesis remained similar in the shallow-light-treated corals compared to the mesophotic-light-treated corals, under both low and high light. However, calcification increased dramatically with increasing light intensity in the shallow-light-treated corals, suggesting a decoupling between these processes. Photoacclimation to shallow-water conditions was indicated by enhanced respiration, a higher density of zooxanthellae per polyp and lower chlorophyll *a* content per cell. The dark-treated corals became completely bleached but did not lower their metabolism below that of the mesophotic-light-treated corals. No *Symbiodinium* clade shift was found following the year-long light treatments. We conclude that *E. paradivisa*, and its original symbiont clade, can adapt to various light conditions by controlling its metabolic rate and growth energy investment, and consequently induce LEC.

## 1. Introduction

Recent studies have demonstrated that coral reefs below recreational SCUBA diving limitation (greater than 30 m depth), commonly referred to as mesophotic coral ecosystems (MCEs), host a thriving community of flora and fauna that has remained almost completely unexplored [1–5]. The MCEs are the deeper environments in the coastal photic zone that separates the shallow reefs from the continental slopes and the aphotic zone [1]. It is assumed that MCE corals occupy at least 50% of the unique potential of coral habitats [6]. In contrast with the shallow reefs (0–30 m depth), where corals are usually exposed to high solar energy (up to 2000 µmol photons m^−2^ s^−1^), MCEs (30–150 m depth) are characterized mainly by a low light intensity (up to 150 µmol photons m^−2^ s^−1^) and narrower (bluish) light spectrum (e.g. [7] and references within). Corals typically found along the shallow reefs have been shown to possess several mechanisms that enable them to cope with the intense light regime: a high density of UV-absorbing mycosporine-like amino acids (MAAs) [8,9], and xanthophylls for non-photochemical quenching [10]. However, very few MCE corals have been examined in this respect [11].

Mesophotic depths in the northern Red Sea are characterized by a lower annual variation in nutrients and temperature than those of the shallows (i.e. [12,13]). Furthermore, seasonal variations in light intensities in the shallow reef can be threefold higher than in the MCEs [14], resulting in constant modification of the photosynthetic apparatus [15,16] in the shallow reef corals.

Some coral species harbour different *Symbiodinium* clades or types across their depth distribution, whereas other corals host a single *Symbiodinium* clade or type across their entire depth range. This adaptation strategy has been shown to have various metabolic advantages [11,17]. In both cases, physiological changes in the holobiont, such as photosynthesis/respiration (*P*/*R*) ratios, become apparent with depth. These changes, however, are assumed to exert a metabolic cost [17]. This raises questions regarding the photoacclimation mechanisms employed by MCE corals if exposed to irradiance beyond the boundary of their distribution [11,17–19].

Surprisingly, unlike shallow corals that are distributed along the upper 30 m, and which cannot survive drastic changes in light intensity [20], no mortality was observed in *E. paradivisa* colonies when transferred from 50 to 5 m depth [14]. The coral *E. paradivisa* is known from highly turbid lagoons in the shallow reefs of the coral triangle [21], but in the northern Red Sea (Eilat) and in high latitude reefs at Okinawa (Japan), it is found in the MCEs only [14]. These two different habitats (i.e. MCEs and turbid lagoons) are both characterized by relatively low light regimes, which allow this species to thrive better than the less adapted species.

Maximizing photosynthesis is also crucial to the coral host because a large portion of its daily carbon requirements derives from the photosynthates translocated from the symbionts [22,23]. One of the most energy-demanding processes in the coral host is the activation of ion transports along the tissue for regulation of pH. Light-enhanced calcification (LEC) is a well-documented phenomenon in hermatypic corals and various other symbiotic marine species, and relates to an increase in calcification rates upon illumination [24]. Although there are several hypotheses regarding the mechanism of LEC, the coupling between LEC and photosynthesis is still not thoroughly understood [25] and has not previously been reported in mesophotic corals. It has been suggested that the uptake of CO_2_ in symbiont photosynthesis may increase the pH of the coral's internal medium and, hence, the carbonate concentration, which in turn enhances precipitation of CaCO_3_ [24,26,27]. It has also been suggested that light influences calcification through an increase in photosynthetic products, specifically fixed carbon [28], O_2_ [29] and ATP [30]. Others, however, have hypothesized that light is directly responsible for enhanced calcification, as the Ca^2+^-ATPase pumps may be responsive to light [31,32]. The extracellular calcifying fluid in the space between the aboral ectoderm and the skeleton displays higher pH during light versus dark [26], mainly due to the intense activity of the Ca^2+^-ATPase pumps [33]. This ion transport process was speculated to form the basis of the LEC of shallow corals, and is assumed to be dependent mostly on the activation of these pumps by means of photosynthetic energy.

Corals at deeper depths may not receive sufficient light to trigger light activation for the LEC process. Consequently, the light habitat of a coral may influence its calcification rates and even its calcification mechanisms, by determining whether the coral will perform LEC or not. Uncovering the calcification rate response to a changing light regime may shed some light on the above hypotheses.

In the present study, specimens of the strictly mesophotic coral *E. paradivisa* were kept ex situ under one of the three light treatments: shallow light, mesophotic light (i.e. their native light conditions) or dark. Following a long-term adaptation to these different light regimes, the corals’ photosynthesis performances, calcification rates and physiological adaptations were assessed. The aim of this study was to determine the photoacclimation capabilities and the coupling between photosynthesis and LEC in a mesophotic species.

## 2. Material and methods

### 2.1. Coral collection and maintenance

*Euphyllia paradivisa* was selected as the model coral for the different light condition experiments because it is abundant in the Gulf of Eilat/Aqaba (GoE/A), found along a broad mesophotic depth gradient (36–72 m depth), easy to fragment into individual polyps and, most importantly, strictly mesophotic in this area [14]. Ten colonies of *E. paradivisa* (*ca* 10 polyps in each colony) were collected from the MCEs offshore the Dekel beach, Eilat, Israel, from 40 to 50 m depth. All 10 colonies were collected under permit 2014/40230 of the Israel Nature and Parks Authority during technical diving. Each colony was placed in a Ziploc bag (SC Johnson, USA) filled with seawater and immediately transferred, in black boxes, to running open-circuit seawater aquaria at the Interuniversity Institute (IUI) in Eilat. Corals were then fragmented into individual polyps and given two months to acclimate under the blue light filter ‘Deep blue’ (Lee filters, UK), which mimics light conditions (0–150 µmol photons m^−2^ s^−1^ and peaks at approx. 450 nm) at *ca* 40 m depth (see electronic supplementary material, figure S1). The day prior to translocation of corals to the experimental light conditions, corals were incubated overnight in 2 mg l^−1^ Alizarin Red-S (Merck KGaA, Germany) solution diluted in seawater for skeleton linear extension estimates [34].

### 2.2. Controlled light experiments

The aim of this experiment was to simulate the physiology of *E. paradivisa* as if growing either at the shallow depth, mesophotic depth or below the photic zone. Both light spectrum and intensity were therefore adjusted in the three treatments in order to mimic the three different environments (electronic supplementary material, figure S1). As we did not measure the absorption spectra of the corals in each treatment, the difference in photosynthetic usable radiation (PUR) between treatments was not calculated. However, the shallow- and mesophotic-light treatments both contain the blue portion of the spectrum, which includes the peak absorption of chlorophyll *a*, but with lower intensities in the mesophotic treatment (electronic supplementary material, figure S1). PUR is, therefore, consequently higher in the shallow-light treatment.

Corals were placed in an open-circuit water system that provides fresh seawater pumped constantly from 30 m depth without filtration (i.e. natural components and phytoplankton flowing from the environment to the system). The water temperature and nutrients were identical to those in natural seawater in the GoE/A and identical in all treatments. No manipulation was performed on the temperature, chemistry or nutrient composition of the seawater, and corals were not artificially fed during the experiment. The first group of corals was exposed to full sunlight, under conditions that are similar to natural sunlight at approximately 3 m depth; the second group was maintained under a blue lighting filter, ‘Deep blue’ (Lee Filters, UK), which mimics approximately 40 m depth light conditions (representing the ‘control’ group); and the third group was kept in a dark room entirely deprived of light. Each polyp was placed inside a separate 2 l glass container with running seawater supply (electronic supplementary material, figure S1). Following 1 year under the above treatments, the dark-treated corals had completely bleached and we could visually observe distinct differences in the size and coral colour between the dark-treated corals and the two other groups. We examined the following physiological parameters in order to determine what had led to these changes: total protein, symbiont density and chlorophyll *a* content, as well as tissue and skeleton volume, mass and density.

Four different colonies’ polyps from each treatment were analysed for the above parameters. Tissue was removed with an airbrush at high air pressure, and the host and zooxanthellae were then separated by centrifuging and homogenizing the tissue, and the zooxanthellae fraction was washed three times with 0.22 µm filtered seawater. Coral tissue and skeleton volumes and mass were determined using the water displacement method [35] before and after tissue removal. Tissue and skeleton densities were calculated according to the tissue and skeleton total volumes and mass. Protein concentrations were determined using Micro BCA protein assay kit (Pierce, Thermo Fisher Scientific, USA) according to the manufacturer's protocol and were normalized to coral tissue volume. Chlorophyll *a* was extracted with 90% acetone and absorbance was measured at 630, 664 and 750 nm with an Ultrospec 2100 *pro* spectrophotometer (Amersham Biosciences, Switzerland). Chlorophyll *a* concentration was determined according to Jeffrey & Humphrey [36] and normalized to zooxanthellae cells. Zooxanthellae cells were counted with a haemocytometer under a microscope and normalized to coral tissue volume.

### 2.3. Calcification and photosynthesis

After 1-year acclimation period under the three light treatments, measurements of calcification and photosynthesis were performed on three polyps from each treatment. Polyps were incubated in sealed 500 ml acrylic metabolic chambers for 2 h during the peak light hours (11.00–14.00 h), while the dark incubation of all corals was done at midnight to avoid diurnal changes in calcification. A full-spectrum metal halide lamp (HQI-BT 400 W, 5000 K, Osram Powerstar) illuminated the corals from above to simulate shallow-light conditions (600 µmol photons m^−2^ s^−1^, e.g. light intensity mean in the shallow reefs in the GoE/A), measured with a LI-190 SA quantum sensor (Li-Cor, Inc.). The mesophotic-light conditions were simulated using the ‘Deep blue’ (Lee filters, UK) filter (i.e. subjecting the corals to blue illumination of 50 µmol photons m^−2^ s^−1^, light intensity mean in the MCEs). The incubation system was thermostatically controlled to match the current seasonal seawater temperature of 24.6°C using a water-bath (RTE 210, Thermo Neslab) and the water was circulated inside the chambers by magnetic stirrers. Water samples were drawn from the chambers at the onset, after 2 h and at the end of each incubation, for analysis of total alkalinity by titration with HCl 0.5 N (800 Dosino, Methrom), Winkler titration (702SM titrino, Methrom) and pH measurements (PHM 93, Radiometer Copenhagen).

The second series of metabolic chamber incubations was performed in a 150 ml acrylic metabolic chamber for measurement of photosynthesis versus irradiance (*P*–*I* curve) at seven light intensities ranging from 0 to 1500 µmol photons m^−2^ s^−1^ of full-spectrum light (electronic supplementary material, figure S2). Light intensity was increased every 20 min and *Δ*O_2_ was measured with ProODO optical dissolved oxygen meter sensors (YSI Inc.). The *P*–*I* curve of each polyp was fitted to a nonlinear hyperbolic tangent function in R software according to the formula of Chalker [37], and the following parameters were calculated: compensation irradiance (*I*_c_); minimum saturating irradiance (*I*_k_), maximum light utilization coefficient (*α*, equivalent to initial slope); and maximum net-photosynthesis rate (*P*_max_).

### 2.4. Identification of algal symbionts

Coral tentacles were sampled for DNA extraction at the end of the 1-year experiment from the shallow-light treatment and mesophotic-light treatment (*n* = 4 for each treatment). Total DNA was extracted with DNeasy Blood & Tissue kit (Qiagen, Germany) according to the manufacturer's protocol using 40 µl of elution buffer. Specific primers (see electronic supplementary material, table S1) for the four main *Symbiodinium* clades in scleractinian corals (i.e. A, B, C and D) were used to identify *Symbiodinium* to the clade level for each sample using PCR as follows: 95°C for 3 min, followed by 34 cycles of 95°C for 30 s, 55°C for 30 s, 72°C for 30 s and a final annealing step at 72°C for 3 min. PCR products were visualized on 2% agarose gel. DNA from isolated algae of the different clades (A–D) was used as a positive control as well as synthetic templates based on available clade sequences

### 2.5. Statistical analysis

Statistical analyses were performed using R software [38] and SigmaPlot 12 (Systat Software). Data were checked for normality (Shapiro–Wilk normality test) and homogeneity of variance (*F*-test) and tested accordingly with ANOVA, non-parametric ANOVA or *t*-test. *Post hoc* tests were done using Tukey's and Dunn's methods. *p*-values of less than 0.05 were considered statistically significant. *P*–*I* curve data parameters were calculated in R, after fitting to nonlinear hyperbolic tangent function (NLS).

## 3. Results

**Figure 1.**
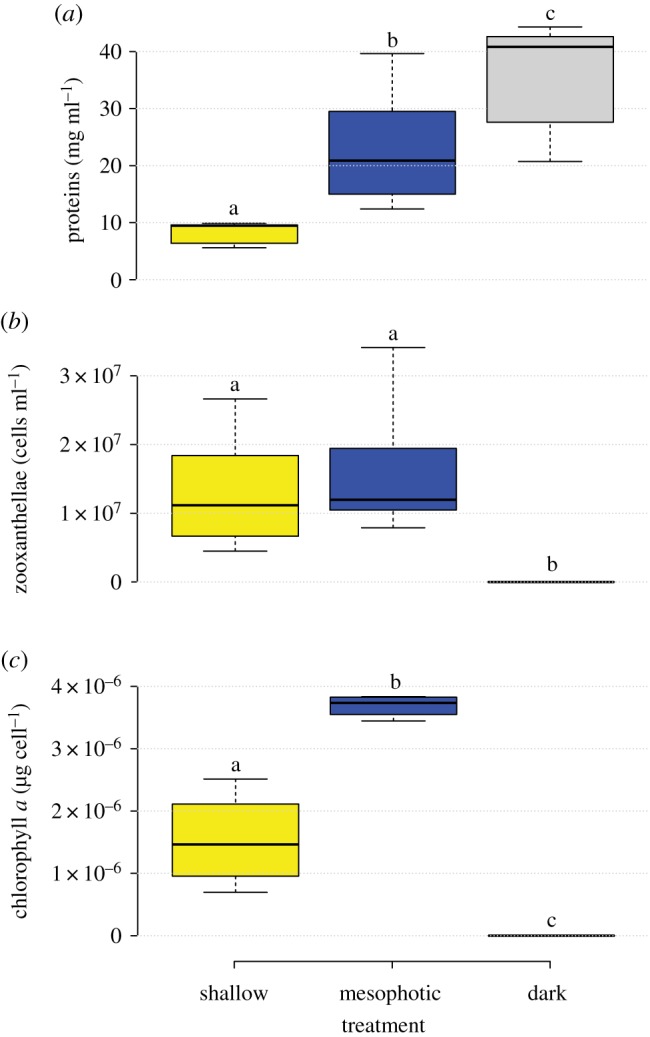
Physiological parameters of the mesophotic coral *E. paradivisa* after 1 year under shallow-light (approx. 3 m), mesophotic-light (approx. 40 m) and dark conditions. (*a*) Total protein concentrations, (*b*) Symbiont density and (*c*) chlorophyll *a* concentrations. Yellow boxes represent shallow-light treatment, blue boxes represent mesophotic-light treatment and grey boxes represent dark treatment; black centred lines represent the medians; box limits indicate the 25th and 75th percentiles; whiskers extend to minimum and maximum values. *n* = 4 polyps sampled from different colonies at each treatment. Letters above the boxes represent statistical significance differences in each parameter.

**Figure 2.**
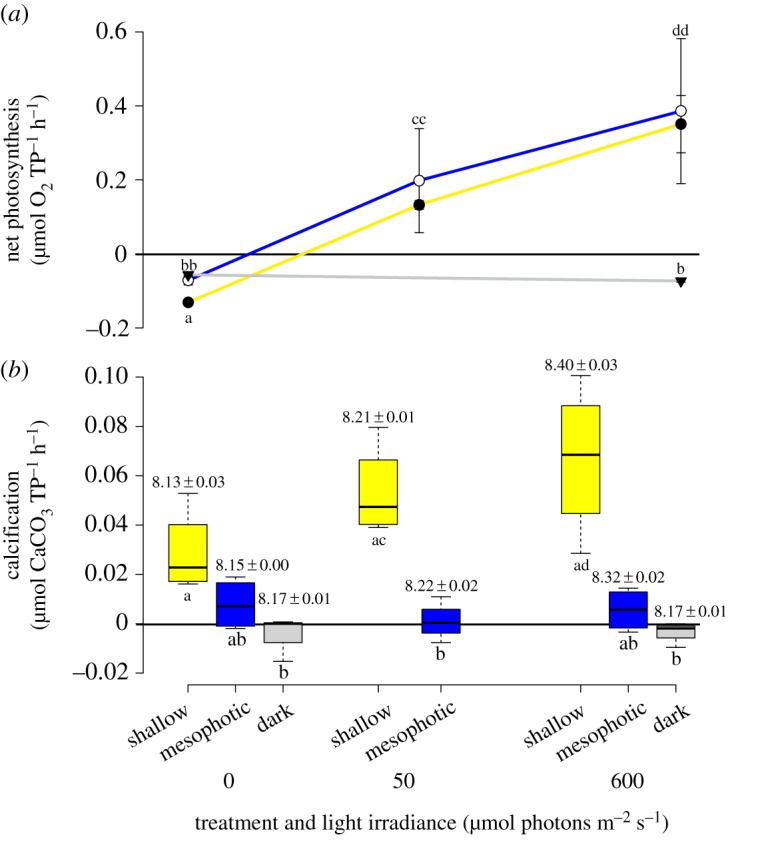
Metabolic chamber photophysiology estimations. (*a*) Net photosynthesis (mean ± s.d.) and (*b*) calcification and pH levels after two incubation hours of *E. paradivisa* under light irradiance of 0, 50 and 600 µmol photons m^−2^ s^−1^. Yellow (shallow-light treatment), blue (mesophotic-light treatment) and grey (dark treatment) represent the 1-year pre-incubation treatment of the corals. Black centrelines in the boxes represent the medians; box limits indicate the 25th and 75th percentiles; whiskers extend to minimum and maximum values; decimal numbers above the boxes represent the post short-incubation seawater pH (mean ± s.d.). *n* = 4 polyps sampled from different colonies at each treatment. Letters above and below the symbols and boxes represent statistical significance differences in each parameter.

Both the host and the symbionts were observed to have modified certain physiological traits following 1 year of exposure to shallow-water conditions in comparison to the mesophotic-light-treated and the dark-treated corals. Compared with the mesophotic-light-treated corals, tissue volume in the dark-treated corals did not change (Tukey's *post hoc* test, *q* = 0.327, *p* > 0.05), but an approximately fivefold increase was observed in the shallow-light-treated corals (table 1). As a result, tissue density in the shallow-light-treated corals had increased significantly (*t*-test, *t* = 2.261, *p* = 0.032) from 0.7 g cm^−3^ in the control to 1.02 g cm^−3^ (e.g. equivalent to the density of the surrounding seawater). Surprisingly, total protein density per millilitre tissue had increased significantly (*t*-test, *t* = 8.472, *p* < 0.001) with decreasing light intensity: that is, the dark-treated corals presented the highest protein concentration (figure 1*a*), whereas respiration was significantly higher (one-way ANOVA, *F* = 62.960, *p* < 0.001) in the shallow-acclimated corals (figure 2*a*), correlating the higher tissue volume. The density of zooxanthellae normalized to tissue volume had not changed significantly after 1 year of exposure to shallow-light (Dunn's *post hoc* test, *Q* = 2.106, *p* > 0.05). However, chlorophyll *a* concentration per zooxanthellae had decreased significantly (*t*-test, *t* = −2.295, *p* = 0.0307) by 60% (figure 1*b*,*c*). We can compare the effect of such modification by comparing photosynthetic rates measured via *P*–*I* curve (electronic supplementary material, figure S2). Up to approximately 80 µmol photons m^−2^ s^−1^, photosynthesis of both shallow-light and mesophotic-light treatments was typical of the light to which they were exposed, i.e. lower *I*_c_ and *I*_k_ in the mesophotic-light corals (table 2; electronic supplementary data, figure S2). However, both treatments corresponded to the higher light intensity of the curve, reaching a similar *P*_max_ per protein (table 2). In the dark-treated corals, no zooxanthellae were found and the tissue was completely bleached, resulting in reduced respiration compared to the shallow-light treatment and no oxygen production (figure 1). This trend was repeated following a longer incubation of 2 h under either low or high light intensity (figure 2*a*). Photosynthesis was normalized to protein in order to assess its impact on calcification. A clear decoupling was observed between these processes when measured for the same incubation: photosynthesis was almost similar for the mesophotic-light and the shallow-light treatments (Mann–Whitney, *T* = 210, *p* = 0.361). However, calcification was significantly higher (Tukey's *post hoc* test, *q* = 8.373, *p* < 0.001) in the shallow-light corals (figure 2*b*). Since photosynthetic rates in both light treatments were similar, seawater pH in the metabolic chambers (noted above the calcification boxes in figure 2*b*) increased with illumination and was also similar between treatments. Calcification has a counter effect on pH as H^+^ is released from $\mathrm{HC}O_{3}^{\;-}$ in the formation of CaCO_3_; however, calcification rates are almost an order of magnitude lower than photosynthesis in the shallow corals (and even more in the mesophotic ones); hence, its effect on the seawater pH is negligible. Furthermore, McConnaughey [39] suggested that these H^+^ ions can be titrated with $\mathrm{HC}O_{3}^{\;-}$ to produce CO_2_ and H_2_O.

**Table 1.** Physiological parameters of the mesophotic coral *E. paradivisa*, after 1 year under shallow-light (approx. 3 m), mesophotic-light (approx. 40 m), conditions and dark conditions. Values are presented as means (±s.d.). Calcification rates were calculated by (SKD)×(LE) for each specimen. *n* = 4–6 polyps sampled from different colonies at each treatment.

treatment	tissue volume (ml polyp^−1^)	tissue density (g cm^−3^)	skeleton density (SKD) (g cm^−3^)	linear extension (LE) (mm year^−1^)	calcification rate (SKD) × (LE) (g cm^−2^ year^−1^)
shallow-light	11.39 (±3.22)	1.02 (±0.07)	1.44 (±0.05)	10.83 (±1.02)	1.56 (±0.58)
mesophotic-light	2.48 (±1.04)	0.70 (±0.28)	1.35 (±0.10)	1.98 (±0.42)	0.27 (±0.14)
dark	2.15 (±0.88)	0.83 (±0.18)	1.36 (±0.02)	1.23 (±0.38)	0.17 (±0.06)

**Table 2.** *P*–*I* curve fitting parameters (graphic illustration in electronic supplementary material, figure S2). Metabolic chamber photosynthesis and respiration measurements of *E. paradivisa* treated with shallow-light (approx. 3 m) or mesophotic-light (approx. 40 m) conditions for 1 year normalized to tissue volume, to total proteins (TP) and non-normalized (representing a single polyp). Parameters were fitted to a nonlinear hyperbolic tangent function: slope, maximum light utilization coefficient (*α*); *R*^2^, coefficient of determination range; *I*_c_, compensation irradiance; *P*_max_, maximum net-photosynthesis rate; *I*_k_, minimum saturating irradiance. *n* = 4 polyps sampled from different colonies at each treatment. s.d., standard deviation of the mean.

	slope	s.d.	*R*^2^	*I*_c_	s.d.	*P*_max_	s.d.	*I*_k_	s.d.
*shallow-light*									
non-normalized	0.115	0.022	0.97–0.99	25.2	6.7	12.9	2.0	115.0	22.4
tissue-normalized	0.010	0.002	0.97–0.99	25.2	6.7	1.2	0.4	115.2	22.6
TP-normalized	0.001	0.000	0.97–0.99	25.2	6.4	0.1	0.0	114.3	22.6
*mesophotic-light*									
non-normalized	0.098	0.007	0.95–0.99	11.0	1.1	6.2	1.2	63.6	16.8
tissue-normalized	0.031	0.007	0.95–0.99	11.1	1.0	1.9	0.1	63.4	16.4
TP-normalized	0.002	0.000	0.95–0.99	10.9	1.0	0.1	0.0	64.2	16.4

**Figure 3.**
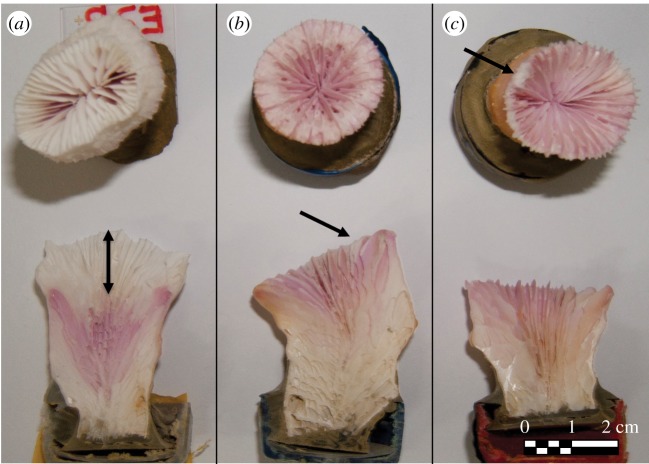
*Euphyllia paradivisa* polyp skeletons after 1 year under (*a*) shallow-light treatment; (*b*) mesophotic-light treatment and (*c*) dark conditions in top-projection view (upper) and cross-section (lower). Arrows show linear extension from the Alizarin Red-S banding.

Despite attaining similar photosynthetic energy and similar oxygen and pH levels, when the different treatments were tested under the same light conditions, corals acclimated to shallow-light conditions (i.e. shallow-light treatment) presented a light-enhanced calcification (LEC) that was not observed in the mesophotic-light corals or in the dark-treated corals. Additionally, these shallow-acclimated corals presented a five to sixfold larger linear extension rate and a much denser septa arrangement in the skeleton (table 1 and figure 3*a*). Moreover, they presented an increased skeleton density starting from 1.35 g cm^−3^ and up to 1.44 g cm^−3^ (table 1; non-significant). When examining the identity of *Symbiodinium* clades, no differences among the three treatments were found. All samples that were analysed at the end of the 1-year exposure to the different light conditions contained clade C *Symbiodinium* symbionts.

## 4. Discussion

The coral *E. paradivisa* is strictly mesophotic in the Red Sea, but can be found at shallower depths in turbid lagoons in the coral triangle [21]. It was shown in a previous study that the Red Sea *E. paradivisa* is able to survive at shallow depths at a high rate [14], even compared to corals that are naturally distributed along the transplantation depth range (i.e. [18,40,41]). We sought to solve this enigma by studying photoacclimation and calcification, and the coupling between these processes, in *E. paradivisa* under shallow depth and dark conditions. Iglesias-Prieto *et al*. [42], who transferred the shallow-coral species *Pavona gigantea* from 10 to 3 m, demonstrated that although photoacclimation was evident, the algal *Symbiodinium* clade did not change. In other corals, bleaching resulting from translocation to the shallow reefs was followed by changes in the symbiont populations [43]. Here, we found a unique response of *E. paradivisa*, 1 year following its transfer from 40–50 to 3 m light conditions: while zooxanthellate density per millilitre of coral tissue did not change significantly, the total number of the zooxanthellate cells per polyp was fivefold higher in the shallow-light treatment than in the mesophotic-light treatment, due to the vast increase in coral tissue volume in the former. Others have previously indicated that zooxanthellae density increases with depth in some corals [16,44] and decreases in others [45–48]. Cohen & Dubinsky [49] correlated this species-specific strategy to the self-shading effect: in shallow corals that generally harbour a low symbiont density (less than 10^5^ cells cm^−2^), the density will increase upon deep acclimation [16,49], while in species harbouring a high zooxanthellae density, the density will decrease with depth [46]. *Euphyllia paradivisa* harboured a high density of symbionts in both the shallow and mesophotic treatments. This could be explained by the ability of this species to mediate the exposure of its long tentacles by means of contraction. Higher zooxanthellae density under high light conditions could lead to a high abundance of reactive oxygen species and, consequently, to bleaching [50,51]. To alleviate this stress, corals possess several mechanisms, such as increasing the activity of catalase and superoxide dismutase [52,53]. In addition, *E. paradivisa* synthesize high levels of fluorescent proteins [54], which are assumed to assist in light dissipation [55]. Similar to other corals [16,46,47,56–60], the amount of chlorophyll per zooxanthellae in this experiment decreased in the shallow-light treatment. However, net photosynthesis, when calculated per protein, was similar in both mesophotic and shallow treatments (electronic supplementary material, figure S2). Nevertheless, the energetic input from photosynthesis is higher in the shallow corals because daily light regimes are closer to their *I*_k_. Dilution of symbionts in the larger tissue volume in the shallow-treated corals, photosynthesis per millilitre coral tissue decreases (electronic supplementary material, figure S2) and correlates to the lower quantum yield of such treatments in a previous study on this species [14]. This modification was not accompanied by a shift in the symbiont population at the clade level (electronic supplementary material, table S1). We found that all samples harboured *Symbiodinium* from clade C, although further investigation might uncover a more subtle change (i.e. at the type level) that was not revealed in the present study. This may correlate with the findings of Lesser *et al*. [19], who showed that the genetic composition of zooxanthellae of the depth-generalist coral *Montastraea cavernosa* shifts below 60 m depth. However, in their study, they found that both morphological and physiological photoacclimation occurred even at a depth of 91 m.

Total protein density per millilitre tissue had increased with decreasing light intensity, while the dark-treated corals presented the highest protein concentration (figure 1*a*). This increase can be explained by higher heterotrophic performance [61] of this group and may indicate storage capacity capabilities of *E. paradivisa* in stress or limited time periods [62]. The increase in skeleton density found for the shallow-light treatment corals may also be related to the changes in morphology that are typical in mesophotic corals in order to minimize light reflectance, compared with shallow corals [63]. Additionally, the higher tissue volume of these corals, combined with higher calcification rates and linear extension, may explain the difference in coral septal density in the shallow-light treatment.

Furthermore, it was surprising that the mesophotic-light-treated corals did not demonstrate any reduction in their O_2_ production rates up to a light intensity of 1500 µmol photons m^−2^ s^−1^, as would have been expected from deep and even shallow corals under this light intensity [32,64]. The effect of photoacclimation was examined here also in regard to LEC (figure 2). The increased metabolism (i.e. higher respiration rates) could explain the increase in dark calcification of corals under the shallow treatment (figure 2*b*). However, while calcification of the high-light-acclimated *E. paradivisa* (i.e. shallow-light treatment) was enhanced by light, which hence performed LEC, as known in shallow corals [28], calcification of *E. paradivisa* under the mesophotic-light or dark treatments took place at similar rates whether short incubated (for 2 h periods) in the dark condition (dark calcification) or under increasing light intensities (figure 2*b*). Obviously, after 1 year, calcification is enhanced in the shallow corals compared with the mesophotic-light and dark-treated corals. This is also seen by the higher extension rates (figure 3) of the shallow corals. From the photosynthetic energetic perspective, although *P*_max_ did not increase upon shallow acclimation, these corals are exposed to irradiance above *I*_k_ throughout most of the day and hence acquire energy that can be directed to calcification (table 2). The proton (H^+^) pumping mechanism hypothesis, recently confirmed by Cai *et al*. [65], describes the energetic cost of calcification, mainly due to the function of ATPase pumps that actively transport ions across tissues, but it might be also the synthesis/secretion of organic matrix molecules [66]. It is not surprising, therefore, that the shallow-light-treated corals acquired higher photosynthetic energy and, as a result, calcified more than the mesophotic-light corals (figure 2; electronic supplementary material, figure S2). However, it is intriguing that LEC occurred only in the shallow treatment even though photosynthesis during the 2 h incubation was similar between treatments (figure 2). Such a decoupling between light calcification and photosynthesis only in deep corals and not in the shallow ones was also observed by Mass *et al*. [60], although *Stylophora pistillata* is not a mesophotic specialist coral. The induction of LEC in mesophotic corals following a 1-year exposure to high irradiance suggests that this mechanism relies on physiological processes (e.g. proton [H^+^] pumps, Ca^2+^-ATPase pumps, organic matrix molecules), rather than solely on the availability of immediate photosynthetic energy. Seawater pH was elevated in the chambers to similar values in both treatments (figure 2*b*), when tested under both low and high light. LEC was thus not a result of photosynthetic CO_2_ uptake, as originally suggested by Goreau [24]. A second LEC mechanism was suggested to depend on a light trigger to upregulate pH and Ca^2+^ levels in the calcifying fluid of corals (e.g. [31]), and therefore related to the function of the Ca^2+^-ATPase pumps. This trigger was suggested to derive from light activation of photoreceptors in the coral tissue due to the correlation between their activation spectrum and LEC [32,67,68]. These photoreceptors are known to synchronize the circadian rhythm and to be stimulated by very low light [69], in order to prepare early on in the day for the impending dramatic light cycle. In preliminary tests, we observed that the *F*_v_/*F*_m_ of *E. paradivisa* was not circadian under either constant light or dark treatment. This may imply that light variation in the mesophotic reefs is so low that such a mechanism is inhibited and also results in the lack of the signalling for Ca^2+^-ATPase pump activation [70].

In previous studies presenting a high-light experimental set-up, we found that although *E. paradivisa* survived well upon translocation to shallow reefs, an established population in the Red Sea was found solely in the MCEs [14,54]. Given that the ‘deep-reef refugia hypothesis' is species-specific [71], we suggest that stressors other than light control the vertical distribution of *E. paradivisa*. One of these could be predation by fishes, as was previously suggested in Eyal *et al*. [14], or the larger temperature fluctuations in the shallow reefs in this region [72].

In conclusion, this study has presented evidence of changes in both the physiological and metabolic activities of the strictly mesophotic *E. paradivisa* coral as a result of changes in the light regime. The corals transferred from a mesophotic environment to shallow-light conditions demonstrated considerable photoacclimation, including LEC (studied for the first time in strictly mesophotic corals) and consequential higher growth rates. By contrast, the mesophotic-light-treated corals were unable to perform LEC during the incubation period of this experiment, and hence are probably unable to do so in nature. The long-term (1 year) lack of photosynthetic energy in the low-irradiance (e.g. the MCEs) environment is probably one of the reasons why these corals were incapable of performing LEC. Additionally, the estimated net calcification rates suggest that the effect of light on calcification rates, whether direct or indirect, might be long term. Furthermore, long-term energetic limitation is suggested to be the main parameter of LEC, and even organisms that do not perform it in one environment may engage this mechanism if supplied with sufficient energy. MCE corals have been shown to experience drastic seasonal variations in the energetic content compared to shallow corals [73]. It is generally accepted that the ecological advantages acquired by corals in their native habitat may jeopardize their survival in cases of abrupt environmental change [74]. However, for the otherwise strictly mesophotic *E. paradivisa* in the Red Sea, shallow waters may be advantageous. The surprising success of *E. paradivisa* in surviving under a full-spectrum shallow-light regime, and hence the potential photoacclimation of strictly MCE corals, calls for further research into these mechanisms. The present results demonstrate that the corals collected from 50 m were able to photoacclimate to the higher light regime at 3 m depth. This suggests that the high illumination at shallow depths is not the only factor that restricts *E. paradivisa* to the MCEs in the Gulf of Aqaba, Red Sea.

## Supplementary Material

Electronic supplementary material (ESM) 1; Dataset in Dryad: https://datadryad.org/review?doi=doi:10.5061/dryad.b6k0275

## Data accessibility

The supporting data are provided in the electronic supplementary material and in the Dryad Digital Repository: http://dx.doi.org/10.5061/dryad.b6k0275 [75].
