# Supplementary material for: Photoacclimation and induction of light-enhanced calcification in the mesophotic coral Euphyllia paradivisa
Source: R Soc Open Sci. 2019 Feb 6;6(2):180527. doi: 10.1098/rsos.180527 (PMC6408387; doi:10.1098/rsos.180527)
Supplement: Electronic supplementary material (ESM) 1; Dataset in Dryad: https://datadryad.org/review?doi=doi:10.5061/dryad.b6k0275 [file rsos180527supp1.pdf]

**<u>Electronic supplementary material (ESM)</u>: Photoacclimation and induction of light-enhanced calcification in the mesophotic coral *Euphyllia paradivisa***

Gal Eyal[1,2,*,#], Itay Cohen[2,3], Lee Eyal-Shaham[1,2], Or Ben-Zvi[1], Yaron Tikochinski[4], Yossi Loya[1]

**<u>Shallow-light</u>** **<u>Mesophotic-light</u>** **<u>Dark</u>**

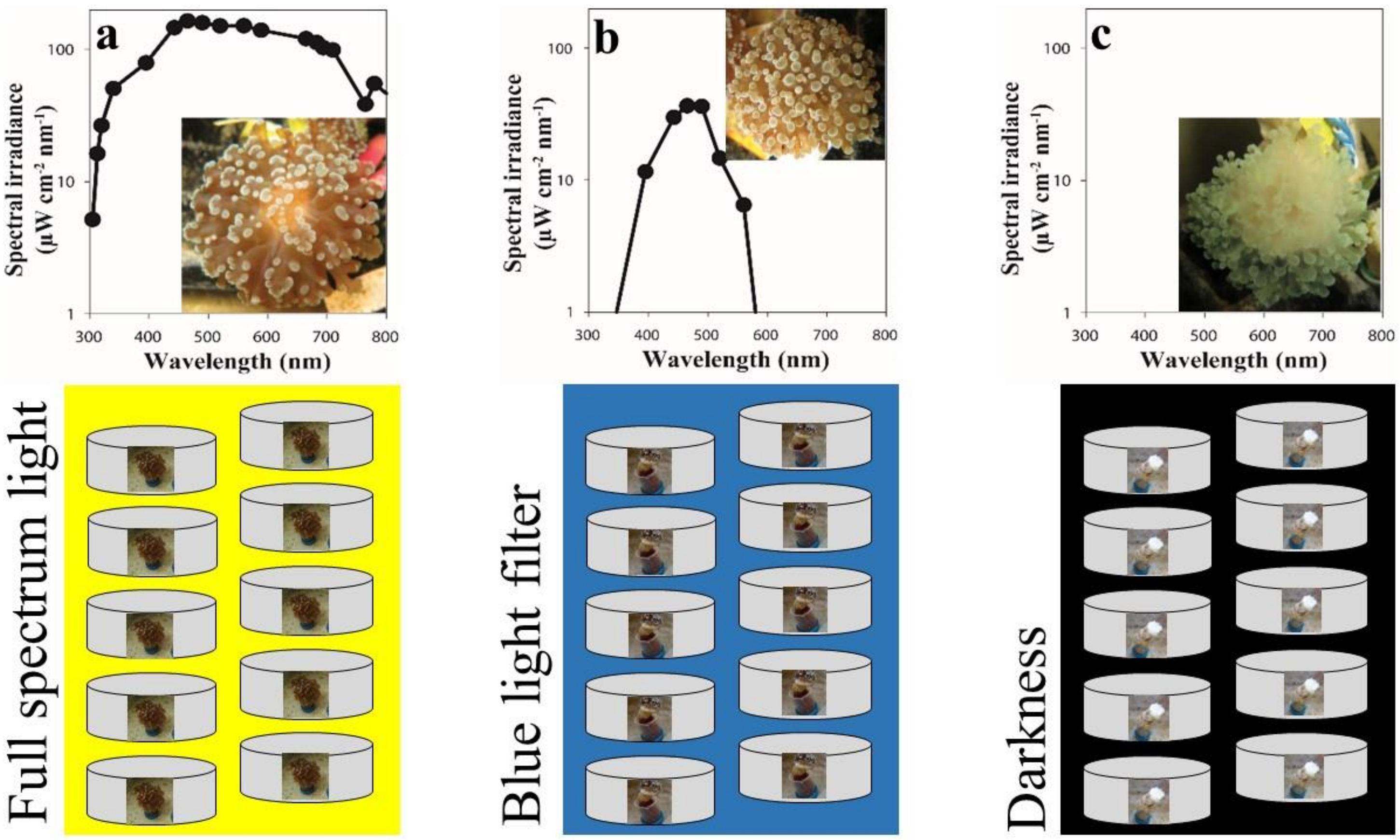


Figure 1s: One-year illustrative experimental set-up of the three different light conditions of the treated *Euphyllia paradivisa*, which simulate; (a) full-spectrum shallow-light, (b) blue mesophotic-light, and (c) deep-sea darkness environments. Each polyp was placed inside a separate two liters glass container with running seawater supply. n = 10 individual polyps in each treatment.

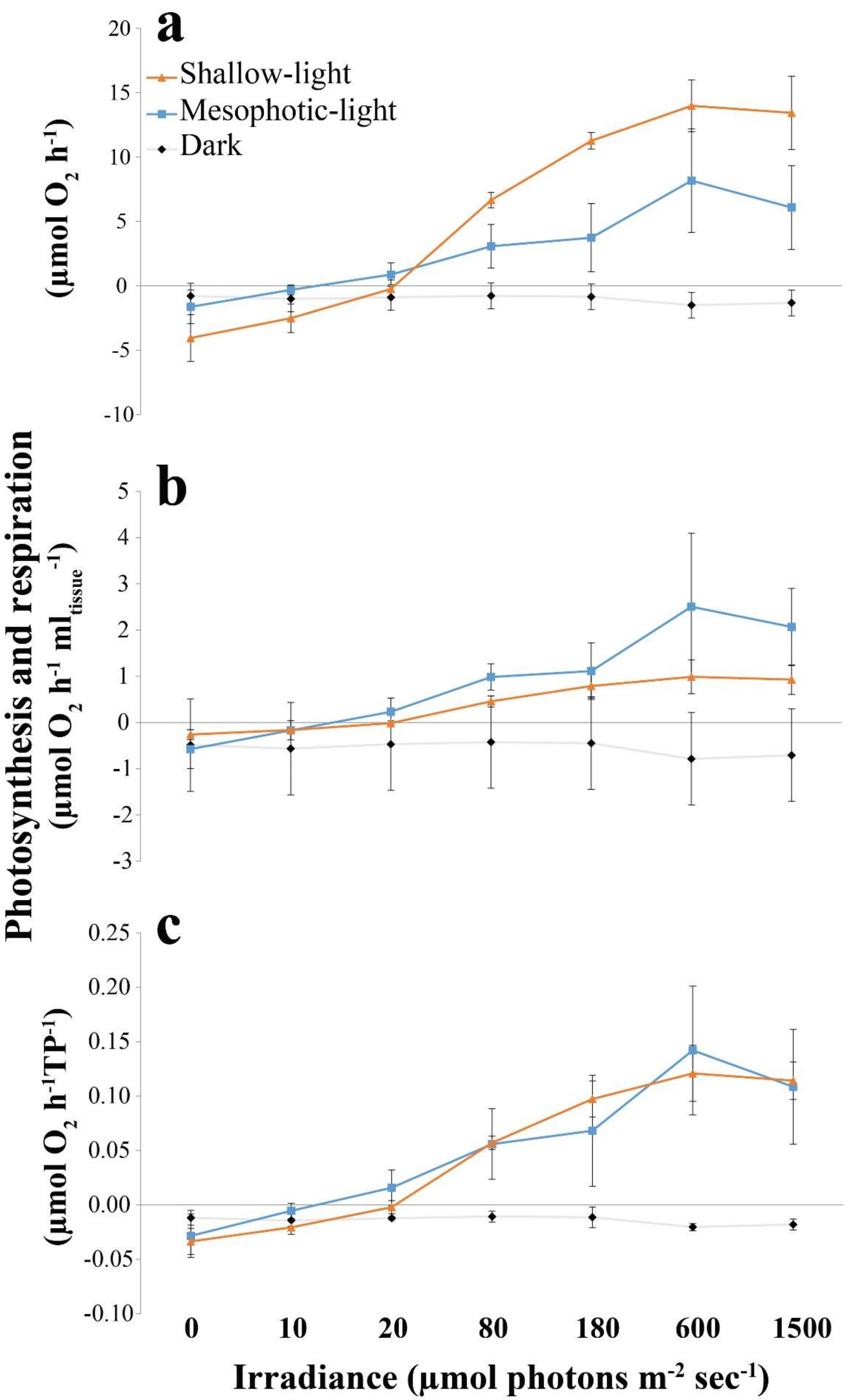
a
Shallow-light
Mesophotic-light
Dark
(µmol O2 h-1)
20
15
10
5
0
-5
-10
b
Photosynthesis and respiration
(µmol O2 h-1 mltissue-1)
5
4
3
2
1
0
-1
-2
-3
c
(µmol O2 h-1TP-1)
0.25
0.20
0.15
0.10
0.05
0.00
-0.05
-0.10
0
10
20
80
180
600
1500
Irradiance (µmol photons m-2 sec-1)

Figure 2s: Photosynthesis vs. irradiance curves (mean±SD) of net photosynthesis of the mesophotic coral *E. paradivisa* recorded in metabolic chambers after one year of incubation under shallow-light treatment, mesophotic-light treatment, simulating light at ~40 m depth), and total darkness. YSI dissolved $O_2$ delta measurements of 20 minutes incubation in metabolic chambers under increased light irradiance of 0 to 1500 µmol photons $m^{-2}$ $s^{-1}$. Orange lines represent shallow-light treated corals (triangles), blue lines represent mesophotic-light treated corals (squares), and grey lines represent dark treated corals (black diamonds). n = 4 individual polyps in each treatment (sampled from different colonies).

Table 1s: Species-specific primers for the four main *Symbiodinium* clades in scelarctinian corals, A, B, C and D that were designed, tested and used to identify the *Symbiodinium* clade in *E. paradivisa*.

| **Clade** | **Primer** | **Sequence** |
|---|---|---|
| A | SITA-405F | 5'-GTGAACCAATGGCCTCTTGA-3' |
| A | SITA-510R | 5'-CCTGATAACAAGAGCAGCAGAA-3' |
| B | SITB-77F | 5'-GGAAGGAGAAGTCGTAACAAGG-3' |
| B | SITB-178R | 5'-CAAGCGTCCCTCACATCAA-3' |
| C | SITC-616F | 5'-GCTTTGCGCGCTGTTATT-3' |
| C | SITC-729R | 5'-ACTTGTCTGACTTCATGCTAGAG-3' |
| D | SITD-18F | 5'-ACTACGGTGAGGGACTGTT-3' |
| D | SITD-109R | 5'-ACAGGCGCGATCGAATATG-3' |
